# Predatory flying squids are detritivores during their early planktonic life

**DOI:** 10.1038/s41598-018-21501-y

**Published:** 2018-02-21

**Authors:** Fernando Á. Fernández-Álvarez, Annie Machordom, Ricardo García-Jiménez, César A. Salinas-Zavala, Roger Villanueva

**Affiliations:** 1 Institut de Ciències del Mar (CSIC), Passeig Maritim, 37-49, E-08003 Barcelona, Spain; 2 Museo Nacional de Ciencias Naturales (MNCN-CSIC), José Gutiérrez Abascal, 2, 28006 Madrid, Spain; 3 Centro de Investigaciones Biológicas del Noroeste (CIBNOR), Mar Bermejo No. 195 Col. Playa Palo de Santa Rita, C.P. 23090 La Paz, Baja California Sur, Mexico

## Abstract

Cephalopods are primarily active predators throughout life. Flying squids (family Ommastrephidae) represents the most widely distributed and ecologically important family of cephalopods. While the diets of adult flying squids have been extensively studied, the first feeding diet of early paralarvae remains a mystery. The morphology of this ontogenetic stage notably differs from other cephalopod paralarvae, suggesting a different feeding strategy. Here, a combination of Laser Capture Microdissection (LCM) and DNA metabarcoding of wild-collected paralarvae gut contents for eukaryotic 18S v9 and prokaryotic 16S rRNA was applied, covering almost every life domain. The gut contents were mainly composed by fungus, plants, algae and animals of marine and terrestrial origin, as well as eukaryotic and prokaryotic microorganisms commonly found in fecal pellets and particulate organic matter. This assemblage of gut contents is consistent with a diet based on detritus. The ontogenetic shift of diet from detritivore suspension feeding to active predation represents a unique life strategy among cephalopods and allows ommastrephid squids to take advantage of an almost ubiquitous and accessible food resource during their early stages. LCM was successfully applied for the first time to tiny, wild-collected marine organisms, proving its utility in combination with DNA metabarcoding for dietary studies.

## Introduction

Cephalopods are active carnivorous predators, with only a few exceptions: *Nautilus* spp. are mainly scavengers and opportunistic predators (e.g.,^1,2^), the vampire squid *Vampyroteuthis infernalis* is a detritivore^3^, and the mesopelagic Ram’s horn squid *Spirula spirula* feeds mainly on detritus and zooplankton^4^. The remaining 845 species described to date^1,5^ are active predators^6^ and their diets are mainly known from studies on their subadult and adult forms. Cephalopods can hatch as large juveniles similar to the adult in morphology, habitat and feeding habits, or may have a less developed planktonic form, known as paralarvae, usually with a different lifestyle than the adults^7,8^. The behavior and diet of cephalopod hatchlings reported to date has demonstrated their active predatory habits from hatching (e.g.,^9,10^), however, this knowledge is mainly based on coastal shallow-water species, due to availability for sampling and laboratory maintenance^11^.

**Figure 1:**
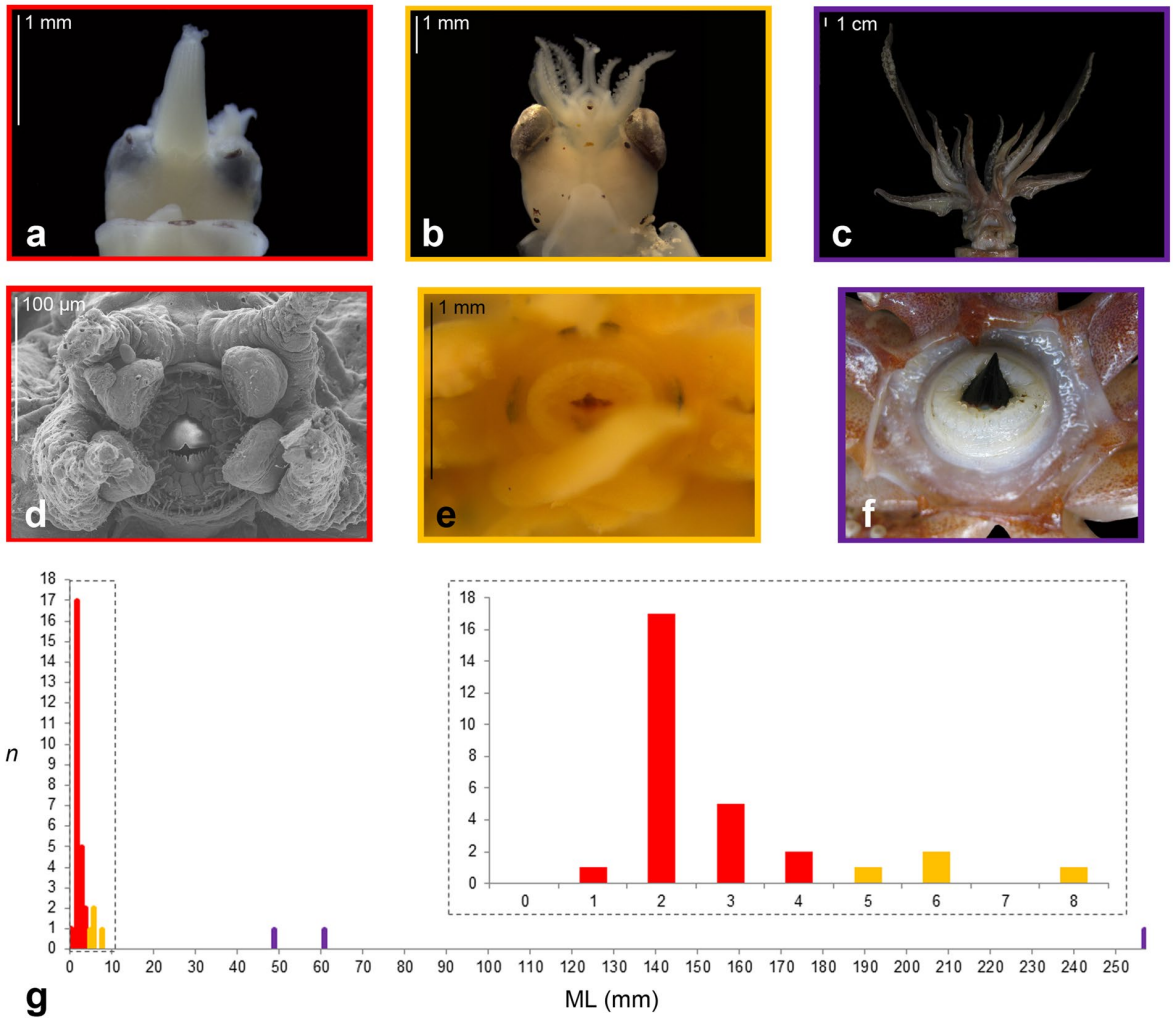
(**a–f**) Morphology of ommastrephid squids. (**a**) Early paralarva (individual E100) showing an unsplit proboscis. (**b**) *Todarodes sagittatus* late paralarva (individual E5) with the proboscis beginning to split. (**c**) Adult *Ommastrephes* sp. individual E3 with the two raptorial tentacles. (**d**) SEM frontal photomicrograph of a *Illex coindetii* early paralarva obtained by *in vitro* fertilization (after Fernández-Álvarez *et al*.,^15^), showing the buccal papillae around the mouth. (**e**) Buccal area of a *Todarodes sagittatus* late paralarva (individual E7). (**f**) Buccal area of a *T. sagittatus* subadult. (**g**) Histogram representing the size classes used in this study, vertical axis represents the number of individuals, the horizontal axis represents the mantle length (mm); red bars represent early paralarvae, while yellow and violet bars represent late paralarvae, and subadults and adults, respectively.

The squid Family Ommastrephidae is currently formed by 22 oceanic species, and represents one of the most widely distributed and ecologically important families of cephalopods^12^. Due to their huge biomass in the oceanic realm, they support some of the largest invertebrate fisheries^13^ and represent nearly 50% of the total cephalopod biomass fished worldwide^14^. The characteristic paralarva of ommastrephids, known as rhynchoteuthion, is characterized by the fusion of both tentacles into a proboscis (Fig. 1a), the function of which is unknown^15^. Along the ontogeny of the squid, the proboscis starts to split^16^ (Fig. 1b) and eventually becomes two independent raptorial tentacles (Fig. 1c), used for prey capture. Newly hatched paralarvae are provided with numerous filamentous buccal papillae around the mouth^17^ (Fig. 1d), which become less abundant as the paralarvae grow until they totally disappear^17^ (Fig. 1e), coinciding with the split of the proboscis into raptorial tentacles^16^ (Fig. 1b,e). The function of these papillae is also unknown. For clarity, throughout the current work, the paralarvae prior to losing the buccal papilla are referred to as “early paralarvae” and after as “late paralarvae”.

The diet of both subadult and adult ommastrephids has been extensively studied (e. g.,^18,19^), however, the diet of the early paralarvae remains unknown^20,21^. All attempts of ommastrephid paralarval rearing have been unsuccessful as paralarvae would not ingest any offered prey^22,23^. Studies on wild caught ommastrephid paralarvae did not provide recognizable prey^20,21^ until the proboscis began to split, the filamentous buccal papillae disappeared and the remains of crustaceans and cephalopods appeared in their stomach contents^20,24^. Interestingly, Vidal and Haimovici^24^ observed a great diversity of microorganisms (dinoflagellates, flagellates, ciliates, cysts and bacteria) on the paralarva mucus cover, on the proboscis suckers and in the digestive tracts of the early ommastrephid paralarvae. They suggested that this mucus may act as a substrate for microbial growth that paralarvae may use this as food and ingest it with the aid of the proboscis. Other authors suggested that ommastrephid paralarvae feed on suspended particles by using the mucus cover of the body^25^, but they did not provide further evidence.

In recent years, studies using molecular tools for identifying gut contents have become more common^26^, especially since Next-Generation Sequencing (NGS) methods became more affordable. Based on this approach, DNA metabarcoding of gut samples is a powerful approach to identify prey remains^27–29^. Particularly, the high number of reads that NGS platforms produce allows the detection of DNA traces or underrepresented prey, highly improving the understanding of the diet of the focal species^28^. Despite these advantages, co-amplification of the target species (self-contamination, hereafter) is an important problem. The key factor is to avoid amplification of the target species, which may be the major^27,29^ or only component of the gut content reads^30^. A number of methods have been selected to overcome this problem, such as the use of primers specific to the prey species^31^. However, this method may only serve to increase the previously extant bias in our knowledge (or belief) about the predator diet^26^ and it cannot be applied when no previous knowledge is available, as in the case of ommastrephid paralarvae. PCR enrichment methods are based on a combination of amplified products with restriction enzymes^32^, DNA blockers^33^ or peptide nucleic acid clamps^34,35^. Nevertheless, Piñol *et al*.^27^ stressed the fact that such blocking molecules are not necessary given the huge number of sequence reads obtained by NGS platforms, which are sufficient to study the diet of focal species even if its DNA co-amplifies.

A critical step that can help diminish self-contamination of the target species is to decrease the amount of predator tissue as much as possible during gut content dissections or extractions. Although this step seems straightforward in large animals, it is not easy to achieve in some tiny organisms, such as small larvae or juveniles of marine animals, which may measure from <1 mm to a few centimeters. Until now, the best dissection method applied to tiny wild-collected marine organisms is syringing of the gut contents^36^. However, the Laser-Capture Microdissection (LCM) method allows the selection of particular tissues or cells from histological sections^37^ and thus, it is a promising method for gut content extraction from tiny animals. Nevertheless, for dietary studies LCM has only tentatively been applied for aquaria-reared cod larvae with a previously known diet, and specific prey primers were applied^38^. Here, we applied LCM gut content dissections in combination with DNA metabarcoding for the first time to assess the first feeding diet of wild-collected ommastrephid squid paralarvae.

## Results

### Taxonomic assignment of eukaryotic reads

Self-contamination reads represented 88.5% of the 2,587,082 reads obtained for 18S v9. Supplementary Fig. S1 represents the percentage of self-contamination and gut contents of each sample successfully sequenced for this molecular marker. For the early paralarvae (n = 25), self-contamination was 78 ± 30% of the reads (range: 0–100%). Four gut samples (E3 to E7) failed to provide any 18S v9 reads matching the SILVA database by the closed-reference approach in Qiime. Whereas the adult individual E3 did not provide any read, the late *T. sagittatus* paralarvae E5 to E7 provided 4,814–208,655 identical sequences that were discarded by the software because they did not match any sequences in the database. A subsequent analysis revealed that these reads matched the 18S v9 Sanger sequenced *T. sagittatus* sequence MF980452, resulting in a self-contamination value of 100%. The LCM-dissected individual E0 was the only late paralarva whose gut contents were successfully sequenced for 18S v9, with a self-contamination percentage of 87.6%. The subadult individuals E1 and E2 were successfully amplified for 18S v9 and showed self-contamination values of 51.0 and 96.7%, respectively.

**Figure 2:**
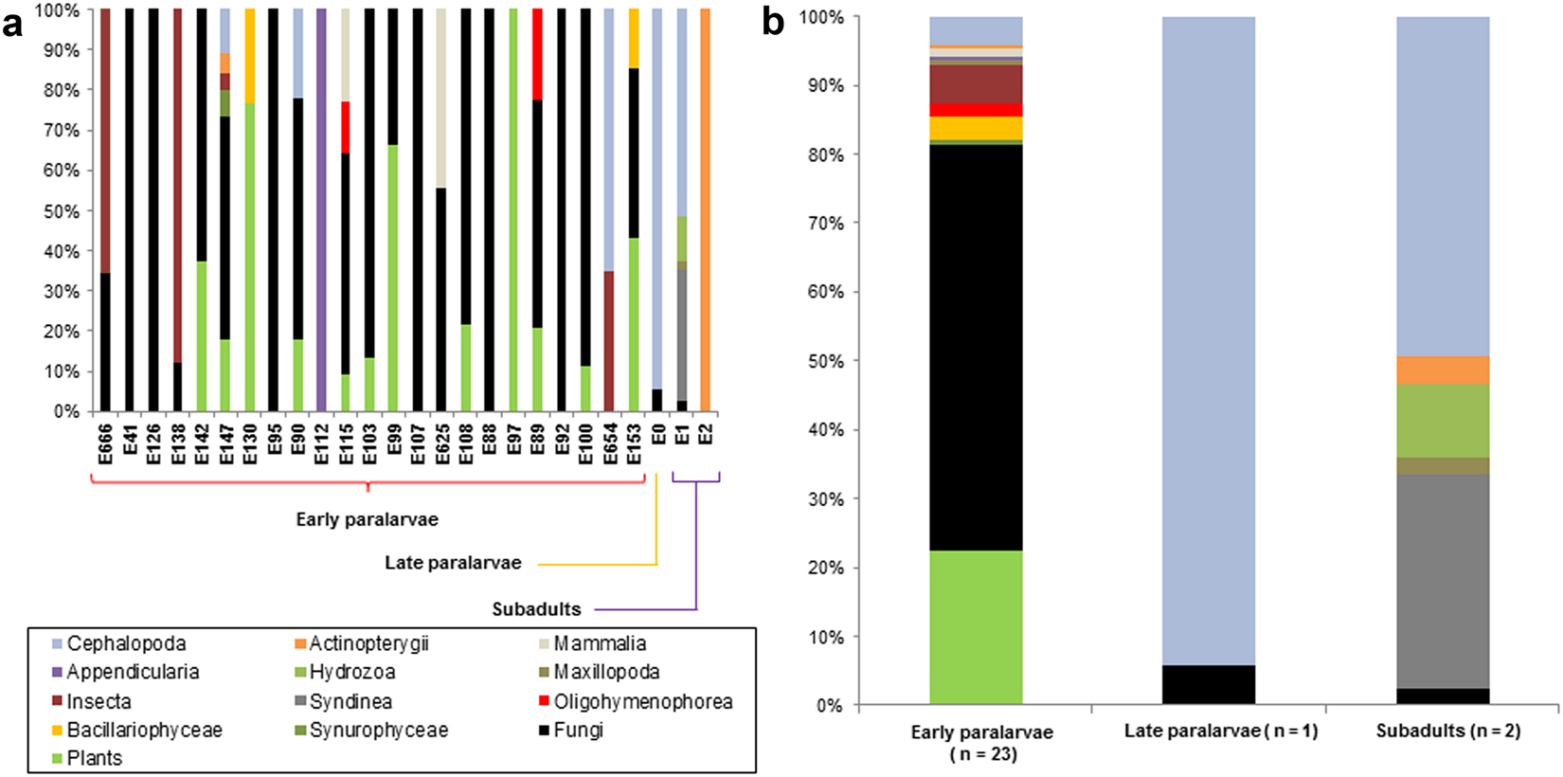
Percentage (%) of eukaryotic 18S v9 reads in the gut contents of each sample (**a**) and grouped by size class (**b**). The taxonomic assignments are at the Class level except plants and fungi, which were collapsed. Self-contamination reads were excluded. Individuals are ordered by mantle length.

After cleaning the self-contamination reads, 299,509 total reads of eukaryotes remained, resulting in 11,519 ± 9,331 (range 1,089–31,566) reads per sample. A total of 59 molecular operational taxonomic units (MOTUs) were identified in the gut contents of all the samples. The percentages of each gut content item of each sample and size class are represented in Fig. 2a and b, respectively, and are summarized by class size in Table 1. The raw gut content reads are available in Supplementary Table S1. Early paralarvae shows 3 ± 2.2 (range 1–11, n = 23) MOTUs, the late paralarva E0 showed 3 MOTUs, and the 2 subadults, 1 and 9 different MOTUs. For the early paralarvae, 22.3% of the gut content reads was composed of plants and 59% was fungi. Animals accounted for 12.6% of the reads, with insects (5.5%) and cephalopods (4.2%) being the most represented groups. The protist groups Chromista and Ciliophora were also present in this size class. The most represented group for the late paralarva and subadults was Metazoa, representing 94 and 66%, respectively. In both the late paralarva and subadults, cephalopods were the most represented group (87.8 and 49.3%, respectively). Parasitic dinoflagellates of the Class Syndinea were only present in the subadult squid E1, representing 31% of the reads of this size class.

**Table 1:** 18S v9 eukaryotic MOTUs detected in the gut contents of ommastrephid squids as a percentage (%) and clustered by size categories.

Kingdom	Phyllum	Class	Early paralarvae (n = 23)	Late paralarva (n = 1)		Subadults (n = 2)	
			Reads (%)	Count	Reads (%)	Reads (%)	Count
Plantae	Magnoliophyta	N/A	3.38	2			
		Eudicotyledoneae	0.69	1			
		Magnoliopsida sp. 1	0.25	1			
		Magnoliopsida sp. 2	0.82	2			
		Magnoliopsida sp. 3	9.95	3			
		Magnoliopsida sp. 4	1.97	2			
		Magnoliopsida sp. 5	1.01	1			
		Magnoliopsida sp. 6	0.47	1			
		Monocotyledoneae sp. 1	0.71	1			
		Monocotyledoneae sp. 2	1.40	1			
		Rosopsida	1.69	3			
Fungi	N/A	N/A	2.71	1			
	Ascomycota	Dothideomycetes sp. 1	5.10	1			
		Dothideomycetes sp. 2	0.33	1			
		Eurotiomycetes sp. 1	4.89	1			
		Eurotiomycetes sp. 2	1.05	1			
		Eurotiomycetes sp. 3	0.29	1			
		Eurotiomycetes sp. 4	2.33	1		2.52	1
		Pezizomycetes	0.33	1			
		Pleosporomycetidae sp. 1	0.42	1			
		Pleosporomycetidae sp. 2	0.64	1			
		Saccharomycetes	12.59	2			
	Basidiomycota	Agaricomycetes sp. 1	4.55	2			
		Agaricomycetes sp. 2	0.27	1			
		Agaricomycetes sp. 3	0.23	1			
		Agaricomycetes sp. 4	1.05	1			
		Basidiomycetes	1.30	1			
		Microbotryomycetes sp. 1	0.49	1			
		Microbotryomycetes sp. 2	2.42	1	5.68		
		Microbotryomycetes sp. 3	0.64	1			
		Microbotryomycetes sp. 4	3.05	4			
		Tremellomycetes sp. 1	8.60	5			
		Tremellomycetes sp. 2	0.56	1			
		Tremellomycetes sp. 3	2.04	1			
		Tremellomycetes sp. 4	0.64	1			
	Entomophthoromycota	Entomophthoraceae	2.51	1			
Chromista	Ochrophyta	Synurophyceae	0.62	1			
		Bacillariophyceae sp. 1	1.78	1			
		Bacillariophyceae sp. 2	1.73	1			
Protista	Ciliophora	Oligohymenophorea sp. 1	1.50	1			
		Oligohymenophorea sp. 2	0.42	1			
	Dinoflagellata	Syndinea sp. 1				2.53	1
		Syndinea sp. 2				4.17	1
		Syndinea sp. 3				3.14	1
		Syndinea sp. 4				21.28	1
Metazoa	Arthropoda	Insecta sp. 1	3.83	1			
		Insecta sp. 2	0.41	1			
		Insecta sp. 3	1.21	2			
		Maxillopoda sp. 1	0.71	1			
		Maxillopoda sp. 2				2.32	1
	Chordata	Appendicularia	0.55	1			
		Actinopterygii sp. 1				4.13	1
		Actinopterygii sp. 2	0.46	1			
		Mammalia	1.16	2			
	Cnidaria	Hydrozoa				10.64	1
	Mollusca	Cephalopoda sp. 1 (*Ommastrephes* sp.)			6.53	6.59	1
		Cephalopoda sp. 2 (*Illex* sp.)				42.68	1
		Cephalopoda sp. 3 (*Sthenoteuthis* sp.)	3.27	2			
		Cephalopoda sp. 4 (*Eucleoteuthis luminosa*/*Dosidicus gigas*)	1.00	2	87.78		
Total reads			261,611		11,541	26,357	

Taxonomic assignments are at a Class level, with the exception of Cephalopoda, which are identified at a genus level. The count number indicates the number of individuals of each class size category with reads for the gut content item. N/A, not applicable.

### Taxonomic assignment of prokaryotic reads

**Figure 3:**
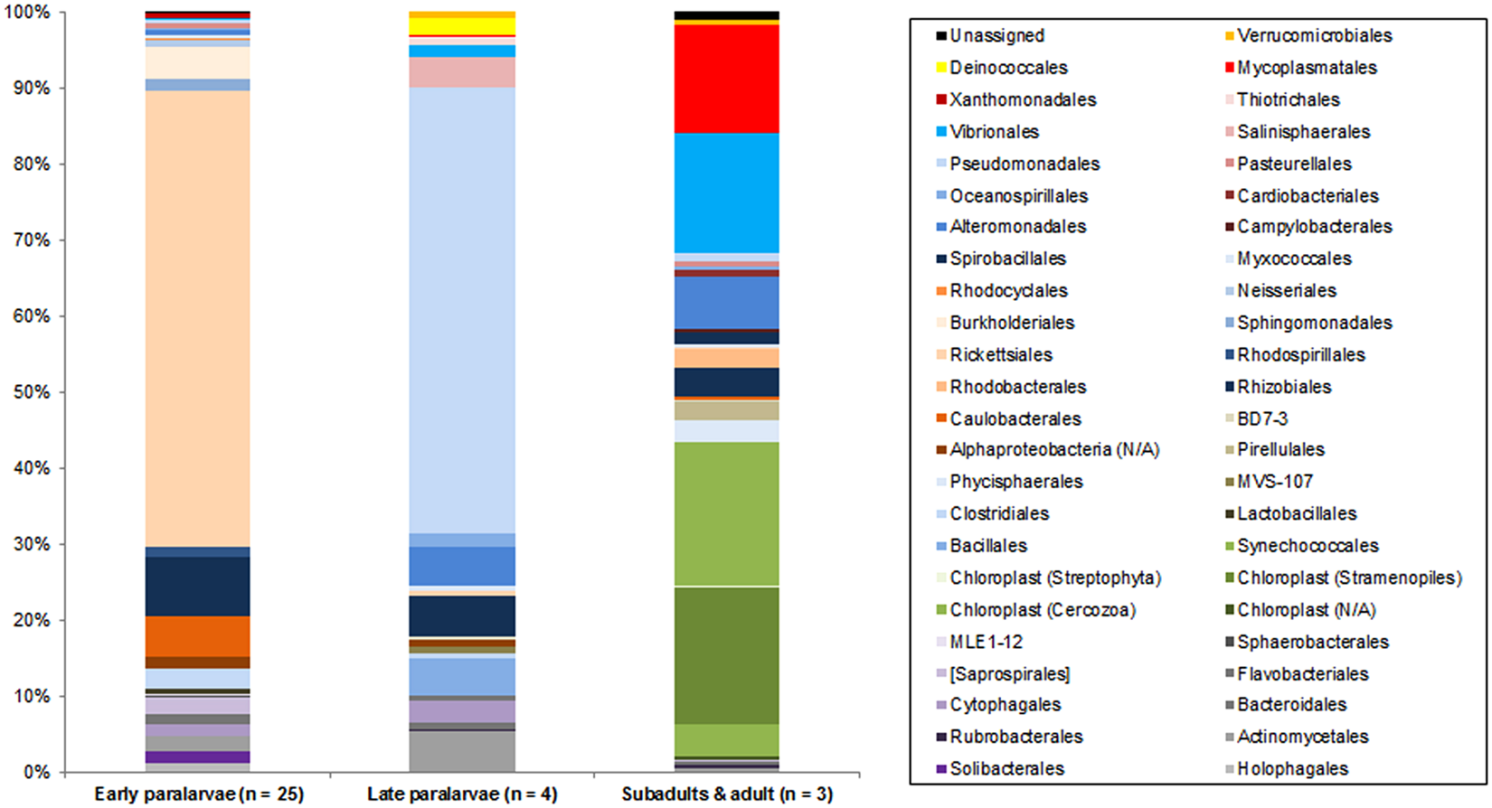
Percentage (%) of the prokaryotic 16S reads in the gut contents grouped by size class. The taxonomic assignments are at the Order level. Chloroplast sequences are eukaryotic chloroplasts amplified with this molecular marker. N/A, not applicable (the finest identification was at the class level).

A total of 453,883 prokaryotic reads were obtained from the gut contents resulting in 14,183 ± 28,280 (range 12–124,004) reads per sample. A total of 141 different MOTUs were identified, with three of them unassigned to any taxonomic level (2,608 reads in total). The percentages of each gut content item at the Order level are represented in Supplementary Fig. S2 for each sample. Percentages of each bacterial Order grouped by size class are represented in Fig. 3 and in Table 2. The raw data are available in Supplementary Table S2. For early paralarvae (n = 25), the most represented group was the Class Proteobacteria (86% of the reads). The Proteobacteria Order Rickettsiales represented 60% of the reads. Some bacterial groups commonly found in Particulate Organic Material (POM), such as Cytophagia, Deltaproteobacteria, Flavobacteria and Firmicutes^39^ were present in early paralarvae gut content. The autotrophic component (Cyanobacteria and chloroplasts) represented 0.3% of the reads of early paralarvae. The Phylum Acidobacteria was only present in early paralarvae (2.6%), while Planctomycetes, present in the other two size categories, was absent in early paralarvae. For late paralarvae (n = 4), the Class Proteobacteria was the most represented group (80%) and the autotrophic component represented 0.05% of the reads. For subadults and the adult (n = 3), Cyanobacteria and chloroplasts were the most represented groups (42%), while Proteobacteria accounted for 35% of the reads and the parasitic Mycoplasmatales for 14%. Since the small size of unicellular Cyanobacteria prevent its selected ingestion by subadults and adults, these sequences can only be explained by the ingestion of food items enriched with these organisms, suggesting predation over herbivores.

**Table 2:** 16S prokaryotic MOTUs detected in the gut contents of ommastrephid squids as a percentage (%) and sorted by size category.

Kingdom	Phyllum	Class	Order	Early paralarvae (n = 25) reads (%)	Late paralarvae (n = 4) reads (%)	Subadults & adult (n = 3) reads (%)
Bacteria	Acidobacteria	Holophagae	Holophagales	1.0354		
		Solibacteres	Solibacterales	1.6018		
	Actinobacteria	Actinobacteria	Actinomycetales	2.0923	5.3699	0.5777
		Rubrobacteria	Rubrobacterales	0.2250	0.2582	
	Bacteroidetes	Bacteroidia	Bacteroidales	0.0005	0.9424	
		Cytophagia	Cytophagales	1.4985	2.7949	
		Flavobacteriia	Flavobacteriales	1.3095	0.7464	0.4268
		[Saprospirae]	[Saprospirales]	2.2642	0.0017	0.3750
	Chloroflexi	Sphaerobacteridae	Sphaerobacterales	0.1870		
	Cyanobacteria	4C0d-2	MLE1-12	0.2908		
		Chloroplast	N/A			0.3389
			Cercozoa	0.0005	0.0051	4.1795
			Stramenopiles	0.0020	0.0119	18.0005
			Streptophyta	0.0010		0.2289
		Synechococcophycideae	Synechococcales	0.0039	0.0375	18.9647
	Firmicutes	Bacilli	Bacillales		4.7189	
			Lactobacillales	0.6359		0.1409
		Clostridia	Clostridiales	2.7018	0.6919	
	Planctomycetes	C6	MVS-107		0.8316	
		Phycisphaerae	Phycisphaerales		2.8827	
		Planctomycetia	Pirellulales		0.0034	2.3244
	Proteobacteria	Alphaproteobacteria	N/A	1.5773	0.8862	
			BD7-3	0.0005	0.6374	0.1713
			Caulobacterales	5.2989	0.0017	0.6002
			Rhizobiales	7.6903	5.1518	3.6583
			Rhodobacterales	0.0005	0.0136	2.4391
			Rhodospirillales	1.3247		
			Rickettsiales	60.1709	0.7601	0.1990
			Sphingomonadales	1.5759		
		Betaproteobacteria	Burkholderiales	4.1440	0.0068	0.1461
			Neisseriales	0.8959		
			Rhodocyclales	0.2286		
		Deltaproteobacteria	Myxococcales	0.5189	0.7004	0.3734
			Spirobacillales		1.4712	
		Epsilonproteobacteria	Campylobacterales	0.0017	0.5651	
		Gammaproteobacteria	Alteromonadales	0.5488	4.9660	6.8196
			Cardiobacteriales	0.0005	0.0034	0.9438
			Oceanospirillales	0.3099	1.7860	0.4667
			Pasteurellales	0.6124	0.0205	0.7228
			Pseudomonadales	0.4773	58.7110	0.9014
			Salinisphaerales	4.0406		
			Vibrionales	0.1601	1.4997	15.8191
			Thiotrichales		1.2066	
			Xanthomonadales	0.6271		
	Tenericutes	Mollicutes	Mycoplasmatales	0.0029	0.1432	14.3777
	[Thermi]	Deinococci	Deinococcales	2.2206		
	Verrucomicrobia	Verrucomicrobiae	Verrucomicrobiales	0.8453	0.4907	
Unassigned	N/A	N/A	N/A	0.2095	0.0170	1.1365
Total reads				204270	58679	190934

Taxonomic assignments are at the Order level. N/A, not applicable.

## Discussion

The mixture of continental (insects, plants and freshwater algae) and exclusively marine animal DNA (appendicularians and cephalopods) in combination with single cell organisms (cyanobacteria, diatoms and ciliophorans), other organisms often associated with organic material degradation (fungi) and bacteria typically associated with POM and fecal pellets, strongly suggest that ommastrephid squids are detritivores during their early planktonic life. Similar assemblages of general gut content composition and protists taxa have been reported in other marine suspension feeders during their larval life, such as eel and spiny lobsters larvae^34,35,40–42^. Interestingly, these gut contents have not been previously reported in the literature for any other cephalopod paralarvae^6,11,21,24,29^. The detritus-based diet of early ommastrephid paralarvae is an unexpected finding, since posterior ontogenetic stages are voracious predators^6,12^. A first feeding diet based on detritus represents a unique life strategy among predatory cephalopods and is potentially one of the reasons for the ecological success of the Family Ommastrephidae in the oceanic realm. This ontogenetic shift in the diet agrees with the change in stable isotopic composition found between ommastrephid early paralarvae and adults in a previous work^43^. A detritivore diet would allow ommastrephid squids to take advantage of an almost ubiquitous and accessible food resource during their early stages, such that they do not directly compete with conspecifics of later ontogenetic stages for the same prey (even if they do predate on different ontogenetic stages of a particular species) or with other cephalopod paralarvae. Since detritus is almost ubiquitous^44–46^, competition for trophic resources between early ommastrephid paralarvae would also be minimal. The new knowledge provided in this work can be applied in the future to the development of experimental culture protocols for ommastrephid hatchlings obtained by *in vitro* fertilization^47^ or aquaria spawning^48^.

Identifying the diet of wild cephalopod paralarvae by DNA sequencing is a poorly studied topic. Only two previous studies exist, both working with coastal species whose paralarvae have a very different morphology and ecology, the common octopus *Octopus vulgaris* and the midsize squid *Alloteuthis media*^29,49^. As far as we know, no previous attempts to study the diet of ommastrephid squids by NGS sequencing have been made. Since no reliable knowledge on the diet of early ommastrephid paralarvae was available, a mixed approach based on sequencing the hypervariable eukaryotic 18S v9 and prokaryotic 16S rRNA was performed here, covering almost every life domain. This combination provided a good snapshot of the diet of early ommastrephid paralarvae. Although more specific eukaryotic metabarcodes are available, the spectrum of taxonomic groups they are able to amplify is usually narrower^50^. Thus, if one of these molecular markers was selected, many eukaryotic MOTUs would not be detected and the study may be critically biased, providing very different results and possibly misleading conclusions.

It should be noted that the bacteria found did not only come from the diet, since gut microbiomes of marine animals are formed by an enormous diversity of bacteria^51^. The cephalopod gut microbiome is poorly understood at present, but has recently gained attention in efforts to overcome mortality problems in laboratory reared paralarvae^52^. To the best of our knowledge, there is no previous work dealing with the gut microbiome of ommastrephid squids, either for paralarvae or later ontogenetic stages. The absence of this information precluded us from reliably distinguishing the bacteria that came from the diet from those that are common residents in the gut microbiome of squids. Similarly, some prokaryotic MOTUs may represent parasites, such as seven MOTUs of Mycoplasmataceae, which represented an important part of the subadult and adult reads (14%, Table 2, Supplementary Table S2). Although the prokaryotic data generated here (Table 2, Supplementary Table S2; Fig. 3 and Supplementary Fig. S2) are in the context of a dietary study, the results provided may aid in the understanding of the gut microbiome of ommastrephids when more directed studies are carried out and may bring to light the possible pathogens that infect these oceanic cephalopods.

**Figure 4:**
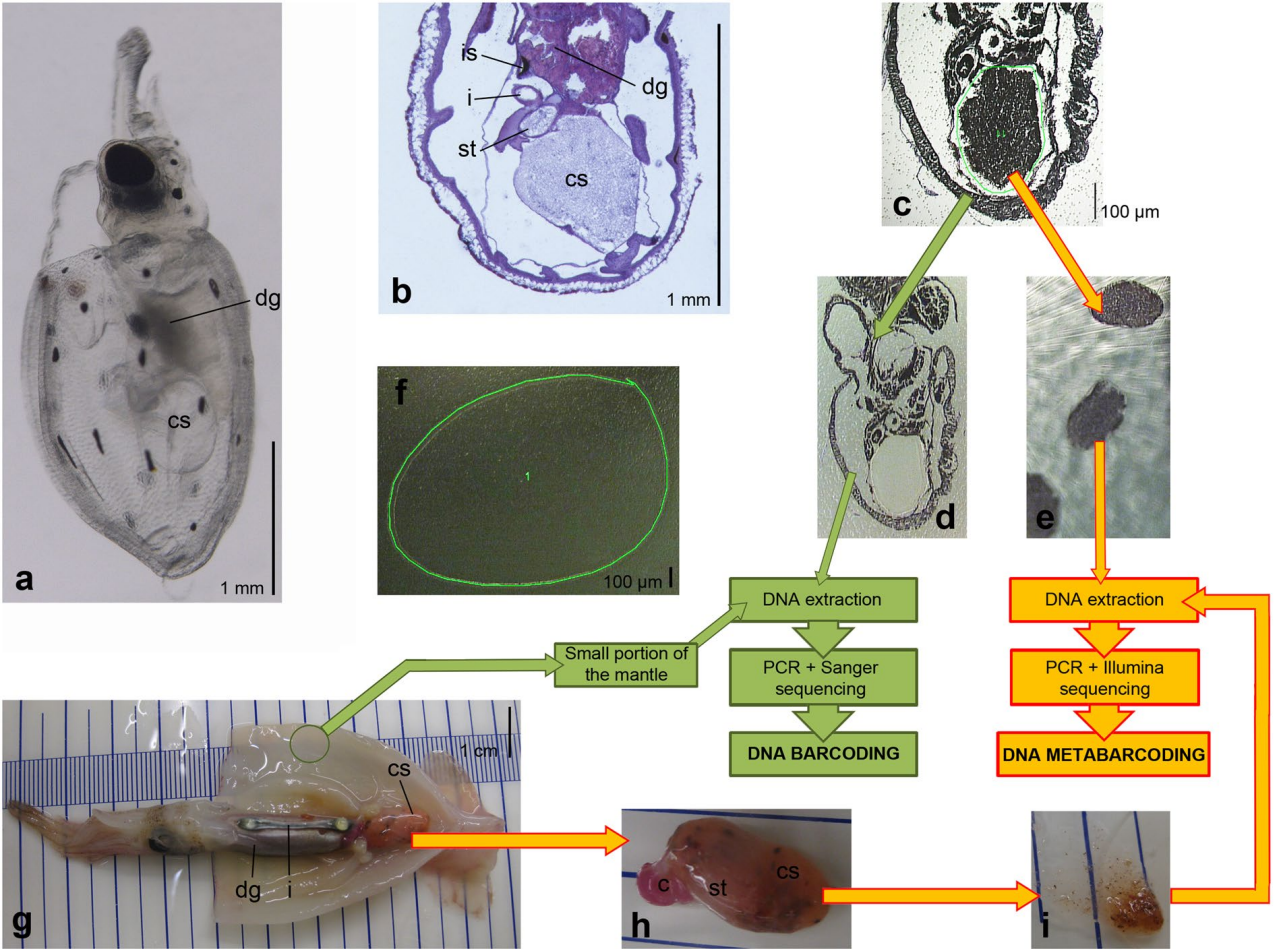
Diagram of the lab workflow. (**a–f**) LCM gut content extraction (late paralarva E0 and early paralarvae, Table 3). (**g**–**i**) Direct dissection of gut contents (subadult and adult individuals E1 to E3 and late paralarvae E5 to E7, Table 3). (**a**) Lateral view of a live hatchling of the ommastrephid squid *Todaropsis eblanae*, obtained by *in vitro* fertilization (after Fernández-Álvarez *et al*.,^15^). (**b**) Histological sagittal section of a *T. eblanae* paralarvae, showing the structure of the digestive system. (**c**) Sagittal section of the early paralarva E41 (*Dosidicus gigas*) mounted on the PEN slide during a LCM session; the green line encircles the area selected for laser cutting. (**d**) Same section as in subfigure C with the caecum sac contents LCM-excised. (**e**) Cuts of LCM-isolated gut contents of several sections of the paralarva E41. (**f**) PEN slide without tissues (blank), the green line shows the portion selected for laser cutting. (**g**) Subadult individual E2 (*Sthenoteuthis pteropus*) with the mantle opened to show the internal organs. (**h**) Caecum sac and caecum of individual E2. (**i**) Isolated gut contents by direct dissection. Abbreviations: c, caecum; cs, caecum sac; dg, digestive gland; i, intestine; is, ink sac; st, stomach.

In this study, gut contents were successfully LCM-isolated from histological paralarvae sections (Fig. 4) and NGS sequencing was carried out with small portions of gut contents (Table 3) obtaining low values of self-contamination (Supplementary Fig. S1). Three late paralarvae were not LCM-processed (E5-E7, Table 3) and the whole digestive system was used in the DNA extraction. Despite the fact that these paralarvae revealed conspicuous gut contents in the digestive system during dissection, no reads were retrieved from the first bioinformatic analysis. A posterior bioinformatic analysis showed 100% self-contamination of *T. sagittatus*, a species not present in the database used (SILVA) (see in Material and Methods). Thus, the only possible explanation is that paralarvae tissues strongly prevail in the PCR product producing 100% of the reads during NGS sequencing. This indicates the importance of avoiding the inclusion of gut tissues from the focal species when performing dietary analyses. The low self-contamination reads obtained in this study for LCM-dissected paralarvae are unusual in the literature of dietary metabarcoding studies of tiny organisms with universal primers and without PCR enrichment methods, which usually show self-contamination values above 90% (e. g.,^27,29^). This is the first time LCM has been applied on wild-collected samples in a dietary study and our results are promising for applying this methodology to other tiny animals, even when universal primers are used without PCR enrichment methods.

**Table 3:** Individuals studied ordered by mantle length (ML).

Labcode	ML (mm)	Species	LCM dissected area (µm^2^)	Approximate weight of dissected gut content (g)	Observations
Early paralarvae					
E666	0.69	*Dosidicus gigas* ^1^	315,441	N/A	Paralarva stage 1
E41	1.02	*Dosidicus gigas* ^1^	1,375,292	N/A	Paralarva stage 1
E126	1.13	SD complex^2^	846,826	N/A	Paralarva stage 1
E138	1.14	SD complex^2^	55,824	N/A	Paralarva stage 1
E142	1.21	*Sthenoteuthis oualanensis* ^1^	1,978,216	N/A	Paralarva stage 1
E147	1.29	SD complex^2^	453,431	N/A	Paralarva stage 1
E130	1.39	*Sthenoteuthis oualanensis* ^1^	219,731	N/A	Paralarva stage 1
E95	1.4	SD complex^2^	2,509,496	N/A	Paralarva stage 1
E90	1.49	*Dosidicus gigas* ^1^	3,033,288	N/A	Paralarva stage 1
E112	1.55	SD complex^2^	1,893,110	N/A	Paralarva stage 1
E115	1.59	SD complex^2^	1,181,529	N/A	Paralarva stage 1
E103	1.64	*Sthenoteuthis oualanensis* ^1^	6,503,474	N/A	Paralarva stage 1
E99	1.67	SD complex^2^	328,239	N/A	Paralarva stage 1
E107	1.74	SD complex^2^	831,532	N/A	Paralarva stage 1
E625	1.88	*Dosidicus gigas* ^1^	3,161,446	N/A	Paralarva stage 1
E108	1.9	SD complex^2^	2,166,047	N/A	Paralarva stage 1
E88	1.91	SD complex^2^	1,328,333	N/A	Paralarva stage 1
E97	1.91	SD complex^2^	483,834	N/A	Paralarva stage 1
E89	2.06	SD complex^2^	3,263,717	N/A	Paralarva stage 1
E626	2.15	*Dosidicus gigas* ^1^	970,475	N/A	Paralarva stage 1
E92	2.17	SD complex^2^	919,236	N/A	Paralarva stage 1
E100	2.29	SD complex^2^	2,432,780	N/A	Paralarva stage 1
E654	2.75	SD complex^2^	4,625,858	N/A	Paralarva stage 2
E153	3.23	SD complex^2^	3,310,476	N/A	Paralarva stage 1
E510	3.75	*Dosidicus gigas* ^1^	6,975,999	N/A	Paralarva stage 1
Late paralarvae					
E6	4.8	*Todarodes sagittatus* ^1^	N/A	No data	Paralarva stage 3
E7	5.2	*Todarodes sagittatus* ^1^	N/A	No data	Paralarva stage 3
E5	5.9	*Todarodes sagittatus* ^1^	N/A	No data	Paralarva stage 3
E0	7.7	*Sthenoteuthis pteropus* ^1^	3,300,000	N/A	Paralarva stage 3
Subadults and adult					
E1	49	*Sthenoteuthis pteropus* ^1^	N/A	0.009	Subadult
E2	61	*Sthenoteuthis pteropus* ^1^	N/A	0.045	Subadult
E3	257	*Ommastrephes* sp.^1,3^	N/A	1.643	Adult male
Extraction blanks					
B1	N/A	N/A	5,415,922	N/A	Extraction blank 1
B2	N/A	N/A	6,343,695	N/A	Extraction blank 2

LCM, Laser Capture Microdissection; N/A, not applicable. Paralarvae stages after Shea^15^.

^1^DNA barcoded individual.

^2^*Sthenoteuthis*/*Dosidicus* species complex: there are no known morphological differences between the two species until *S. oualanensis* paralarvae develop their photophores (ca. 4 mm ML).

^3^*Ommastrephes bartramii* is a species complex according to Fernández-Álvarez *et al*.^65^ although the genus is currently considered monotypic^12^. We avoided providing a species-level identification until its taxonomic status is resolved.

## Material and Methods

### Sample collection

In total, 32 individuals were analyzed: 25 early paralarvae, 4 late paralarvae, 2 subadults and an adult (Table 3, Supplementary Table S3). Further information on sample collection and species identification is available in Supplementary Methods.

### Gut contents extraction

Two methods were developed to extract the gut contents of the individuals according to their size: LCM for small paralarvae and direct dissections for larger squids (Fig. 4). Further information is available in Supplementary Methods.

### DNA extraction

The DNA from the gut samples obtained by LCM dissections was extracted using the QIAamp DNA Investigator Kit (Qiagen) following the corresponding manufacturer’s protocols, and “Isolation of Total DNA from Tissues” for the remaining samples (Table 3). The samples were eluted twice in 30 µl and the second elution was stored at −20 °C as a back-up. Ambient contamination was avoided as much as possible working in the “Ancient DNA lab” of the Museo Nacional de Ciencias Naturales (MNCN-CSIC, Madrid, Spain), isolated from the other rooms and provided with UV light sterilization. Beyond the usual measures to avoid contamination in a molecular systematics lab, additional measures to avoid ambient contamination were: (1) the whole laboratory was cleaned and UV-sterilized before starting the work, and (2) no additional people were working in the same lab during the DNA extraction session.

For molecular identification of the individuals, the remaining tissues on the PEN slides (Fig. 4d) of the LCM-dissected paralarvae and a small portion of the mantle (Fig. 4g) of the remaining squids were dissected with a sterile blade. DNA was extracted using the BioSprint 15 DNA kit (Qiagen), following the manufacturer’s protocol.

### DNA metabarcoding of gut contents

DNA extractions of gut contents were used to construct two libraries, for eukaryotic and prokaryotic DNA identification, covering almost all life domains. This strategy was selected due to of the absence of reliable knowledge of the actual diet of ommastrephid paralarvae. Further information on library preparation and sequencing is available in Supplementary Methods.

### Bioinformatic analysis

The quality of the FASTQ files was checked using the software FastQC^53^ and the Illumina-specific adapters were trimmed by running the cut adapter tool implemented in Trimmomatic^54^. The sequences were quality-filtered (minimum Phred quality score of 20) and labeled using Qiime 1.9.0^55^. The paired-end assembly of forward (R1) and reverse (R2) reads was executed in FLASH^56^ implemented in Qiime. The mismatch resolution in the overlapping region was accomplished by keeping the base with the higher quality score. Artifacts such as point mutations and chimaeras were detected and deleted using the UCHIME algorithm^57^ implemented in VSEARCH^58^. Using the final list of representative sequences, each molecular operational taxonomic unit (MOTU) was searched against the reference database SILVA^59^ v. 128 (September, 2016) and the last available version (May, 2013) of Greengenes^60^ for the 18S v9 and 16S databases, respectively. The 18S v9 reads were clustered into MOTUs using the closed-reference approach with the UCLUST algorithm^61^ in Qiime with a similarity threshold of 100%. Sequences that did not provide a 100% match were discarded. The 16S reads were clustered using the open-reference approach with a similarity threshold of 97% and reads that did not hit the reference sequence collection were subsequently clustered de novo. After this step, singletons and sequences representing less than 0.005% of the total number of sequences of each dataset were excluded. Sequences were compared with the GenBank database by BLAST^62^. For 16S, 30% of the reads remain unidentified using only Greengenes as a database. Thus, BLAST hits were applied to identify at the lowest taxonomic level possible following the same criteria: (1) sequences with <90% for identity or coverage were not considered; (2) 97% similarity is considered the species-level threshold; (3) when more than one sequence has the same identity value, the one identified to the lowest taxonomic level was selected; (4) if the GenBank identification differs at the genus level to that of Greengenes, the latter is applied. Before applying the BLAST identifications, only 0.6% of the sequences were unidentified.

In DNA metabarcoding studies, the mistagging phenomenon has been reported^63,64^, in which a low percentage of the reads of a sample can be assigned to another as the result of the misassignment of the indices during library preparation, sequencing, and/or demultiplexing steps. To correct for this phenomenon, for 18S v9 the low abundance MOTUs of each sample were removed by applying a threshold based on the presence of mistagging in the PCR negative control (i.e., the higher number of reads for a particular sequence in the PCR blank), resulting in a particular threshold for each sequence. As a result, for 18S v9 no sequences were assigned to three of the late paralarvae (individuals E5 to E7) and the adult (individual E3). For 16S, the 0.005% threshold was selected according to the presence of low abundance MOTUs in the whole dataset. Although extraction measures for avoiding ambient contamination were applied, some MOTUs were present in the extraction blanks. Any sequence present in at least one of these blank samples was taken as ambient contamination and removed from the study. For 18S v9, the identifications were performed at the Class level, since some Orders of some of the Classes (e.g., Mammalia and Actinopterigii) could not be reliably assessed with this region. It should also be noted that several related species may share the same metabarcode^50^ and thus, the number of actual eukaryotic species inside the gut may be larger than the number of detected MOTUs. Taxonomic assignments of 16S reads were considered as species-level identifications. Rarefaction plots of each sample were constructed showing the rarefied number of MOTUs defined at 100 and 97% similarity thresholds for 18S v9 and 16S, respectively (Supplementary Fig. S3).

The selected primers for 18S v9 can amplify ommastrephid squid DNA. Thus, although the dissection methodology was performed carefully, DNA of the paralarvae or the squids may be present among the gut contents and must be considered as self-contamination. In a first step, all the 18S v9 ommastrephid sequences available in GenBank were downloaded. An additional *Todarodes sagittatus* sequence obtained with the primers Euk-B and 18_v9_Con following the PCR conditions explained above and Sanger sequenced (GenBank accession number MF980452) was added. All of these sequences were aligned and the *p*-distance percentages were calculated to determine if there were sufficient differences to distinguish these species with this molecular marker (Supplementary Table S4). For those paralarvae successfully identified with DNA barcoding (Table 3), the reads that matched their identification at a genus level were regarded as self-contamination and the others as a component of the diet. For paralarvae that were not molecularly identified and had reads for only one ommastrephid species, the reads were considered as self-contamination reads. If an unidentified paralarva had sequences for two genera, the most represented was regarded as self-contamination and the other as gut contents. The self-contamination component of the reads is expressed as a percentage of the individual reads in relation to the total number of reads obtained for the sample.

Once the self-contamination reads were identified, they were discarded and the remaining reads were analyzed. The percentage of each gut content item was calculated in relation to the number of total sequences of each sample without the self-contamination reads.

### Data Availability

DNA sequences from ommastrephid 18S v9 and 16S gut content and COI sequences of barcoded ommastrephids were deposited in GenBank with the accession numbers MF980393-MF980451, MF980453-MF980593 and MF980594-MF980608, respectively. All data generated and analyzed during this study are included here or as Supplementary Information files.

## Electronic supplementary material

Supplementary material

Supplementary Table S1

Supplementary Table S2
