# Supplementary material for: Predatory flying squids are detritivores during their early planktonic life
Source: Sci Rep. 2018 Feb 21;8:3440. doi: 10.1038/s41598-018-21501-y (PMC5821876; doi:10.1038/s41598-018-21501-y)
Supplement: Supplementary file 1 — Supplementary material [file 41598_2018_21501_MOESM1_ESM.pdf]

Supplementary Material for

Predatory flying squids are detritivores during their early planktonic life

Fernando Á. Fernández-Álvarez[1,2], Annie Machordom[2], Ricardo García-Jiménez[2], César A. Salinas-Zavala[3], Roger Villanueva[1]

[1]Institut de Ciències del Mar (CSIC), Passeig Maritim 37-49, E-08003 Barcelona, Spain; [2]Museo Nacional de Ciencias Naturales (MNCN-CSIC), José Gutiérrez Abascal, 2 28006 Madrid, Spain; [3]Centro de Investigaciones Biológicas del Noroeste (CIBNOR), Mar Bermejo No. 195 Col. Playa Palo de Santa Rita, C.P. 23090 La Paz, Baja California Sur, México.

Corresponding author: f.a.fernandez.alvarez@gmail.com

Supplementary Material and Methods

Sample collection

Wild-collected ommastrephid paralarvae (n = 25) from the northeast Pacific were sampled using a Bongo net (500 µm) during four oceanic cruises (Table S3). The samples of these four oceanic cruises were directly fixed in 70-96% ethanol. The remaining individuals were collected in the central Atlantic under the MAFIA cruise during April 2015 using the methodology described by Olivar et al.[1]. Additionally, a nearly mature *Ommastrephes* sp. was fished by jigging during the MAFIA cruise. All the samples from the MAFIA cruise were frozen on board at -20 ºC until they reached the lab. The mantle or the gut contents were then directly fixed in 96% ethanol. Table 3 summarizes the information for each individual and the sampling location coordinates are available in Table S3. Cephalopod paralarvae are usually fixed in formalin in most oceanographic surveys (e.g., [2],[3]). Thus, available paralarvae suitable for DNA extraction are scarce. In order to overcome this problem and represent the entire ommastrephid life cycle as much as possible, we sampled available specimens, which belonged to different species and had different origins (Table 3, Supplementary Table S3). All ommastrephid paralarvae share the same specialized morphological characters of the mouth, tentacles and arm crown and all the previously examined species also share the same ontogenetic shift in their diet (see [4],[5]). Thus, it is expected that the diet is similar to the same ontogenetic stage in each ommastrephid species.

DNA barcoding for squid identification

Each individual was identified at the finest taxonomic level possible by morphological characters and this identification was molecularly corroborated by amplifying a region of the cytochrome c oxidase subunit I (COI), as a DNA barcode[6]. The squids sampled included almost every ontogenetic phase of the ommastrephid life cycle after hatching, from a putative hatchling (individual with the labcode E666, Table 3), bearing only 1 sucker on arms I and II (see [3]), to an almost mature male (individual E3, Table 3). The size of the individuals used for this study is depicted in Figure 1G. The paralarvae stages were classified according to the criteria proposed by Shea[7] (Table 3). For the dietary analyses, three different size classes were considered to cluster the samples: early paralarvae (0.6-3.8 mm ML, n = 25, paralarvae stages 1-2 *sensu* Shea[7]), late paralarvae (4.8-7.7 mm ML, n = 4, paralarvae stage 3 *sensu* Shea[7]) and subadults and adults (49-257 mm ML, n = 3).

We amplified sequences from the partial COI gene, using the primer pair LCO1490[8] and COI-H[9]. All PCRs were performed in a total volume of 25 µl that included 0.2 µl of MyTaq polymerase (5 U/µl,

Bioline), 5 µl of MyTaq reaction buffer, 0.5 µl of each primer (10 µM), and 2 µl of template DNA. PCRs consisted of an initial denaturation at 95 ºC (1 min), followed by 5 cycles of denaturation at 95 ºC (15 s), annealing at 40 ºC (90 s) and extension at 72 ºC (1 min) and 35 cycles with the same conditions, but with 44 ºC as annealing temperature. The post-PCR products were purified with ExoSAP-IT (Thermo Fisher) prior to sequencing both strands on an ABI Prism 3730 (Applied Biosystems).

The taxonomic identification of the individuals is indicated in Table 3. The morphological identification of the *Sthenoteuthis pteropus* late paralarva coded as E0, the *S. pteropus* subadults E1 and E2, and the *Ommastrephes* sp. adult individual E3 were molecularly confirmed. The late paralarvae E5 to E7 were successfully molecularly identified as *T. sagittatus*. Regarding the early paralarvae, six individuals (labcodes E41, E90, E510, E625, E626 and E666) were identified as *Dosidicus gigas* and another three (E103, E130 and E142) as *Sthenoteuthis oualanensis*. The remaining paralarvae were preliminarily identified after Fernández-Álvarez et al.[3] on the basis of the proboscis suckers as Type C paralarvae, which has proboscis suckers all of the same size. In north Pacific waters, the Type C paralarvae can belong to *S. oualanensis* or *D. gigas*. Until *S. oualanensis* develops ocular and visceral photophores at 4 mm mantle length (ML), there are no known morphological differences between *D. gigas* and *S. oualanensis*[10] and these paralarvae are commonly referred to in the literature as the SD complex (e. g., [11]). Thus, 16 of the studied paralarvae were classified as members of the SD complex.

Gut contents extraction

The gut contents of the paralarvae were isolated by LCM using a Leica LMD 6000. The whole paralarva or its mantle (depending on the size) was placed on standard histological cassettes and embedded in paraffin following the Peterfi method[12]. The paraffin blocks were stored at -20 ºC until histological processing. Each sample was serially sectioned at 10 µm and sections were mounted on Leica nuclease-free polyethylene naphthalate (PEN) membrane slides (PEN slides, hereafter). After mounting, slides were air-dried at room temperature and stored at -20 ºC until staining with hematoxylin-eosin. Several measures were performed to avoid any possible ambient contamination: (1) each reagent was new; (2) every lab tool (microtome, blades, histological hand tools, gloves and nuisance face mask) was cleaned and UV-sterilized at the beginning of each lab session and between each sample; (3) the slides were covered with a UV-sterilized box during drying; (4) a nuisance face mask was used during sectioning to prevent contamination from breathing; (5) the staining protocol was carried out in a laminar flow hood; (6) no additional people were working in the same lab during the histology procedures. Moreover, two paraffin blocks containing no sample were processed as controls following the same methodology as the samples (B1-B2, Table 3) in order to identify any possible ambient contamination during lab sessions.

After the drying stage, the PEN slides were stored at -20 ºC until the LCM sessions. The caecum sac of the paralarvae is lined with a short epithelium (Figure 4B) and is usually full of contents, simplifying the LCM gut content extraction. Thus, the caecum sac was the structure selected to extract the gut contents during the LCM sessions. Another advantage is that this part of the digestive system occupies a medial position, posterior to the esophagus, digestive gland and stomach (Figure 4A-B,G-H), preventing possible bias due to food ingestion inside the fishing net. All laser excisions were performed at 10x magnification and catapulted into sterile 0.2 ml microcentrifuge tubes. For each paralarva, the excised area (Figure 4E) was annotated (Table 3) as a proxy for the amount of gut contents used in each DNA extraction. The samples were stored at -20 ºC until the DNA extraction. The PEN slides with the remaining tissues of the paralarvae (Figure 4D) were also stored at -20 ºC for their molecular identification. Portions of the extraction blanks were LCM-excised (Figure 4F) following the same protocol.

Samples of the subadult and adult individuals were directly dissected (Figure 4G-I). The caecum and caecum sac were carefully dissected (Figure 4H) and the gut contents (Figure 4I) transferred to a pre-tared sterile Petri dish and weighed. Approximately one-third of the gut content was fixed in 96% ethanol for DNA extraction (Table 3). Of the other two-thirds, one was used for DNA extraction probes and the other was fixed in 4% seawater formalin as a morphological voucher of the gut contents. A small portion of the mantle was preserved in 96% ethanol for molecular corroboration of the morphological

identification. The remaining whole body of the squids was fixed in 4% buffered formalin and transferred to the Biological Reference Collections of the Institut de Ciències del Mar (CBR-ICM, Barcelona, Spain) as morphological vouchers under the accession numbers ICMC000057-ICMC000059 (individuals E1 to E3, respectively, Table 3). In order to test the effect of the efficiency of the LCM, the late paralarvae labeled with the codes E5 to E7 (Table 3) were directly dissected instead of LCM-processed, and the whole digestive system of each individual was dissected and used for the DNA extraction.

DNA metabarcoding of gut contents

For eukaryotic DNA, the universal primers Euk-B[13] and 18s_v9_Con[14], which amplify a ~105-165 bp fragment of the hypervariable v9 region of 18S rRNA, were selected. These primers have the advantage of amplifying almost every eukaryotic organism (i.e., animals, plants, fungi, algae, etc.) and the amplicons obtained are small enough to amplify highly digested DNA. In addition, 18S RNA is a multicopy gene, which also increases the possibility of amplifying prey. However, this primer versatility comes at the price of taxonomic resolution[15] and identification to the species level is frequently not possible[16,17]. For prokaryotic DNA, a ~200-210 bp region of the 16S rRNA was amplified with the universal primers 16S-F and 16S-R[18].The PCRs for both fragments were performed in a total volume of 25 µl, adding 1 µl of template DNA, 0.5 µM of the selected primers, and 12.5 µl of Phusion DNA polymerase mix (Thermo Scientific). The reaction conditions were 98 ºC for 30 s, followed by 35 cycles of 98 ºC for 10 s, 60 ºC for 20 s, 72 ºC for 20 s, and a final extension step at 72 ºC for 10 minutes. Strict precautions were taken to avoid environmental contamination during the PCRs, including: 1) the use of a laminar flow hood previously treated with UV light, 2) only filter pipette tips were used, and 3) all surfaces were periodically wiped with bleach. A second PCR round with identical conditions and only 5 cycles were performed for attaching the index sequences. A negative control without DNA was added to check for contamination during library preparation. Libraries were purified using the Mag-Bind RXNPure Plus magnetic beads (Omega Biotek) following the manufacturer´s protocol. They were then quantified with the Qubit dsDNA HS Assay (Thermo Fisher) and pooled in equimolar amounts (10 nM). The pool was sequenced in ½ of a MiSeq paired-end 300 bp run (Illumina).

Supplementary references

1 Olivar, M. P. *et al.* Mesopelagic fishes across the tropical and equatorial Atlantic: Biogeographical and vertical patterns. *Prog. Oceanogr.* **151**, 116-137, doi:10.1016/j.pocean.2016.12.001 (2017).

2 Zaragoza, N. *et al.* Effects of contrasting oceanographic conditions on the spatiotemporal distribution of Mediterranean cephalopod paralarvae. *Hydrobiologia* **749**, 1-14, doi:10.1007/s10750-014-2132-x (2014).

3 Fernández-Álvarez, F. Á., Martins, C. P. P., Vidal, E. A. G. & Villanueva, R. Towards the identification of the ommastrephid squid paralarvae (Mollusca: Cephalopoda): morphological description of three species and a key to the north-east Atlantic species. *Zool. J. Linnean Soc.* **180**, 268–287, doi:10.1111/zoj.12496 (2017).

4 Vidal, E. A. G. & Haimovici, M. Feeding and the possible role of the proboscis and mucus cover in the ingestion of microorganisms by rhynchoteuthion paralarvae (Cephalopoda: Ommastrephidae). *Bull. Mar. Sci.* **63**, 305–316 (1998).

5 Uchikawa, K., Sakai, M., Wakabayashi, T. & Ichii, T. The relationship between paralarval feeding and morphological changes in the proboscis and beaks of the neon flying squid *Ommastrephes bartramii*. *Fish. Sci.* **75**, 317–323, doi:10.1007/s12562-008-0036-2 (2009).

6 Hebert, P. D. N., Cywinska, A., Ball, S. L. & deWaard, J. R. Biological identifications through DNA barcodes. *Proc. R. Soc. Lond. B Biol. Sci.* **270**, 313–321, doi:10.1098/rspb.2002.2218 (2003).

7 Shea, E. K. Ontogeny of the fused tentacles in three species of ommastrephid squids (Cephalopoda, Ommastrephidae). *Invertebr. Biol.* **124**, 25–38, doi:10.1111/j.1744-7410.2005.1241-04.x (2005).
8 Folmer, O., Black, M., Hoeh, W., Lutz, R. & Vrijenhoek, R. DNA primers for amplification of mitochondrial cytochrome c oxidase subunit I from diverse metazoan invertebrates. *Mol. Marine Biol. Biotechnol.* **3**, 294–299 (1994).
9 Machordom, A., Araujo, R., Erpenbeck, D. & Ramos, M. A. Phylogeography and conservation genetics of endangered European Margaritiferidae (Bivalvia: Unionoidea). *Biol. J. Linnean Soc.* **78**, 235–252, doi:10.1046/j.1095-8312.2003.00158.x (2003).
10 Vecchione, M. Extraordinary abundance of squid paralarvae in the tropical eastern Pacific Ocean during El Niño of 1987. *Fish. Bull.* **97**, 1025–1030 (1999).
11 Aceves-Medina, G., Silva-Dávila, R. D., Cruz-Estudillo, I., Durazo, R. & Avendaño-Ibarra, R. Influence of the oceanographic dynamic in size distribution of cephalopod paralarvae in the southern Mexican Pacific Ocean (rainy seasons 2007 and 2008). *Lat. Am. J. Aquat. Res.* **45**, 356-369, doi:10.3856/vol45-issue2-fulltext-11 (2017).
12 Pantin, C. F. A. *Técnicas microscópicas para zoólogos.* (Editorial Academia León, 1968).
13 Medlin, L., Elwood, H., Stickel, S. & Sogin, M. The characterization of enzymatically amplified eukaryotic 16S-Iike rRNA-coding regions. *Gene* **71**, 491–499, doi:10.1016/0378-1119(88)90066-2 (1988).
14 O'Rorke, R. *et al.* Determining the diet of larvae of western rock lobster (Panulirus cygnus) using high-throughput DNA sequencing techniques. *PLoS ONE* **7**, e42757, doi:10.1371/journal.pone.0042757 (2012).
15 Leray, M. & Knowlton, N. Censusing marine eukaryotic diversity in the twenty-first century. *Philos. Trans. R. Soc. Lond. B Biol. Sci.* **371**, 20150331, doi:10.1098/rstb.2015.0331 (2016).
16 O'Rorke, R., Lavery, S. D., Wang, M., Nodder, S. D. & Jeffs, A. G. Determining the diet of larvae of the red rock lobster (Jasus edwardsii) using high-throughput DNA sequencing techniques. *Mar. Biol.* **161**, 551-563, doi:10.1007/s00227-013-2357-7 (2013).
17 Albaina, A., Aguirre, M., D., A., Santos, M. & Estonba, A. 18S rRNA V9 metabarcoding for diet characterization: a critical evaluation with two sympatric zooplanktivorous fish species. *Ecol. Evol.* **6**, 1809–1824, doi:10.1002/ece3.1986 (2016).
18 Reddy, B. V. *et al.* Natural product biosynthetic gene diversity in geographically distinct soil microbiomes. *Appl. Environ. Microbiol.* **78**, 3744-3752, doi:10.1128/AEM.00102-12 (2012).
19 Fernández-Álvarez, F. Á. *et al.* The genus *Ommastrephes* d'Orbigny, 1834: a single species or more than one hidden behind a single name? in *CIAC 2015* (Hakodate, Japan, 2015).
20 Jereb, P. & Roper, C. F. E. Cephalopods of the world. An annotated and illustrated catalogue of cephalopod species known to date. Vol. 2 (FAO, 2010).

Table S3. Collection data of the individuals used in this work. Individuals are ordered by their ML.

Labcode	Species	DML (mm)	Geographical coordinates	Depth (m)	Seafloor depth (m)	Date	Hour	Cruise
**Early paralarvae**								
**E666**	*Dosidicus gigas*†	0.69	25º 45´N, 113º 27´W	0-300	3347	9.2.2005	17:14	IMECOCAL-CICESE, Pacific cruise
**E41**	*Dosidicus gigas*†	1.02	27º 51´N, 112º 17´W	0-150	684.5	26.6.2008	22:33	DGGOLCA, Pacific cruise
**E126**	SD complex ‡	1.13	18º 28´N, 106º 9´W	0-150	>1500	18.1.2010	11:38	PCM, Pacific cruise
**E138**	SD complex ‡	1.14	18º 0´N, 105º 26´W	0-150	>1500	18.1.2010	4:08	PCM, Pacific cruise
**E142**	*Sthenoteuthis oualanensis*†	1.21	18º 0´N, 105º 26´W	0-150	>1500	18.1.2010	4:08	PCM, Pacific cruise
**E147**	SD complex ‡	1.29	18º 0´N, 105º 26´W	0-150	>1500	18.1.2010	4:08	PCM, Pacific cruise
**E130**	*Sthenoteuthis oualanensis*†	1.39	18º 0´N, 105º 26´W	0-150	>1500	18.1.2010	4:08	PCM, Pacific cruise
**E95**	SD complex ‡	1.4	17º 31´N, 104º 54´W	0-150	>1500	17.1.2010	20:56	PCM, Pacific cruise
**E90**	Dosidicus gigas†	1.49	18º 52´N, 105º 5´W	0-150	>1500	15.1.2010	22:44	PCM, Pacific cruise
**E112**	SD complex ‡	1.55	17º 31´N, 104º 54´W	0-150	>1500	17.1.2010	20:56	PCM, Pacific cruise
**E115**	SD complex ‡	1.59	17º 31´N, 104º 54´W	0-150	>1500	17.1.2010	20:56	PCM, Pacific cruise
**E103**	*Sthenoteuthis oualanensis*†	1.64	17º 31´N, 104º 54´W	0-150	>1500	17.1.2010	20:56	PCM, Pacific cruise
**E99**	SD complex ‡	1.67	17º 31´N, 104º 54´W	0-150	>1500	17.1.2010	20:56	PCM, Pacific cruise
**E107**	SD complex ‡	1.74	17º 31´N, 104º 54´W	0-150	>1500	17.1.2010	20:56	IMECOCAL-CICESE, Pacific cruise
**E625**	*Dosidicus gigas*†	1.88	24º 39´N, 114º 2´W	0-300	No data	3.8.2005	3:32	IMECOCAL-CICESE, Pacific cruise
**E108**	SD complex ‡	1.9	17º 31´N, 104º 54´W	0-150	>1500	17.1.2010	20:56	PCM, Pacific cruise
**E88**	SD complex ‡	1.91	18º 59´N, 104º 28´W	0-150	259	21.1.2010	19:19	PCM, Pacific cruise
**E97**	SD complex ‡	1.91	17º 31´N, 104º 54´W	0-150	>1500	17.1.2010	20:56	PCM, Pacific cruise
**E89**	SD complex ‡	2.06	18º 52´N, 105º 5´W	0-150	>1500	15.1.2010	22:44	PCM, Pacific cruise
**E626**	*Dosidicus gigas*†	2.15	24º 39´N, 114º 2´W	0-300	No data	3.8.2005	3:32	IMECOCAL-CICESE, Pacific cruise
**E92**	SD complex ‡	2.17	19º 18´N, 107º 18´W	0-150	>1500	19.1.2010	6:25	PCM, Pacific cruise
**E100**	SD complex ‡	2.29	17º 31´N, 104º 54´W	0-150	>1500	17.1.2010	20:56	PCM, Pacific cruise
**E654**	SD complex ‡	2.75	25º 45´N, 113º 27´W	0-300	878	9.2.2005	0:11	IMECOCAL-CICESE, Pacific cruise
**E153**	SD complex ‡	3.23	17º 31´N, 103º 44´W	0-150	>1500	16.1.2010	17:45	PCM, Pacific cruise
**E510**	*Dosidicus gigas*†	3.75	26º 9´N, 114º 7´W	0-300	No data	31.7.2005	16:53	IMECOCAL-CICESE, Pacific cruise
**Late paralarvae**								
**E6**	*Todarodes sagittatus*†	4.8	21º 36´N, 18º 55´W	0-50	2989	25.4.2015	4:56	MAFIA, Atlantic cruise
**E7**	*Todarodes sagittatus*†	5.2	21º 36´N, 18º 55´W	0-50	2989	25.4.2015	4:56	MAFIA, Atlantic cruise
**E5**	*Todarodes sagittatus*†	5.9	21º 36´N, 18º 55´W	0-50	2989	25.4.2015	4:56	MAFIA, Atlantic cruise
**E0**	*Sthenoteuthis pteropus*†	7.7	7º 9´N, 23º 58´W	0–100	4245	17.4.2015	23:28	MAFIA, Atlantic cruise

**Subadults and adult**								
**E1**	*Sthenoteuthis pteropus*†	49	3º 45´N, 25º 15´W	0-800	4170	15.4.2015	21:47	MAFIA, Atlantic cruise
**E2**	*Sthenoteuthis pteropus* †	61	3º 45´N, 25º 15´W	0-800	4170	15.4.2015	21:47	MAFIA, Atlantic cruise
**E3**	*Ommastrephes* sp.† §	257	18º 7´N, 20º 11´W	No data	3174	23.4.2015	23:05	MAFIA, Atlantic cruise

† DNA barcoded individual.

‡ *Sthenoteuthis/Dosidicus* species complex: there are no known morphological differences between both species until *S. oualanensis* paralarvae develop their photophores (ca. 4 mm ML).

§ *Ommastrephes bartramii* is a species complex according with Fernández-Álvarez et al.[19] although the genus is currently considered monotypic[20]. We avoided providing a species-level identification until its taxonomic status is solved.

Table S4. Uncorrected *p*-distances (%) of 18S v9 sequences of ommastrephid squids.

Sthenoteuthis oualaniensis (AY557511)	*S. oualaniensis*	*E. luminosa*	*D. gigas*	*O. bartramii*	*T. sagittatus*
Eucleoteuthis luminosa (EU735294)	1.4				
Dosidicus gigas (KY387931)	1.4	0			
Ommastrephes bartramii (AY557510)	2.1	2.1	2.1		
Todarodes sagittatus (MF980452)	4.1	2.8	2.8	2.1	
Illex coindetii (AY557509)	2.8	2.8	2.8	3.4	2.8

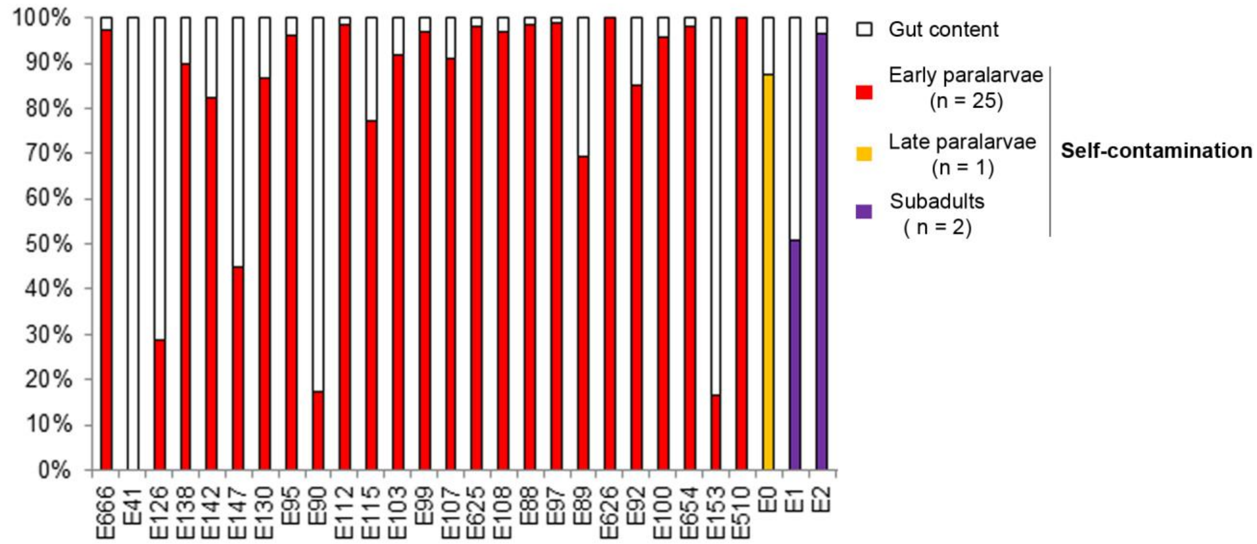

Figure S1. Percentage of self-contamination (solid color) found in the 18S v9 metabarcoding gut content analysis. Individuals are ordered by mantle length.

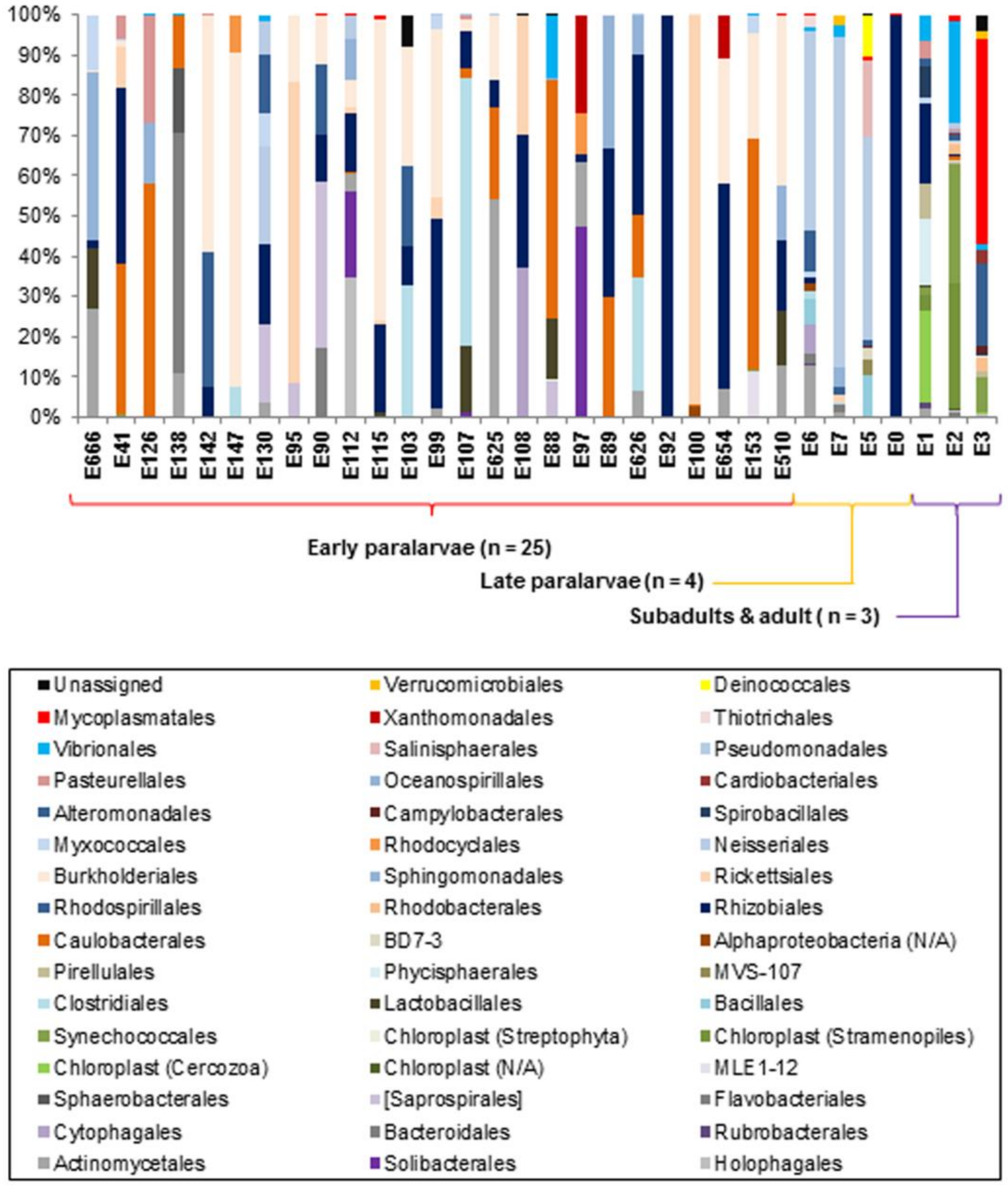

Figure S2. Percentage (%) of the prokaryotic 16S reads in the gut contents of each sample. The taxonomic assignments are at the Order level. Individuals are ordered by mantle length. Chloroplast sequences are eukaryotic chloroplasts amplified with the 16S primers. N/A, not applicable (the finest identification was at the class level).

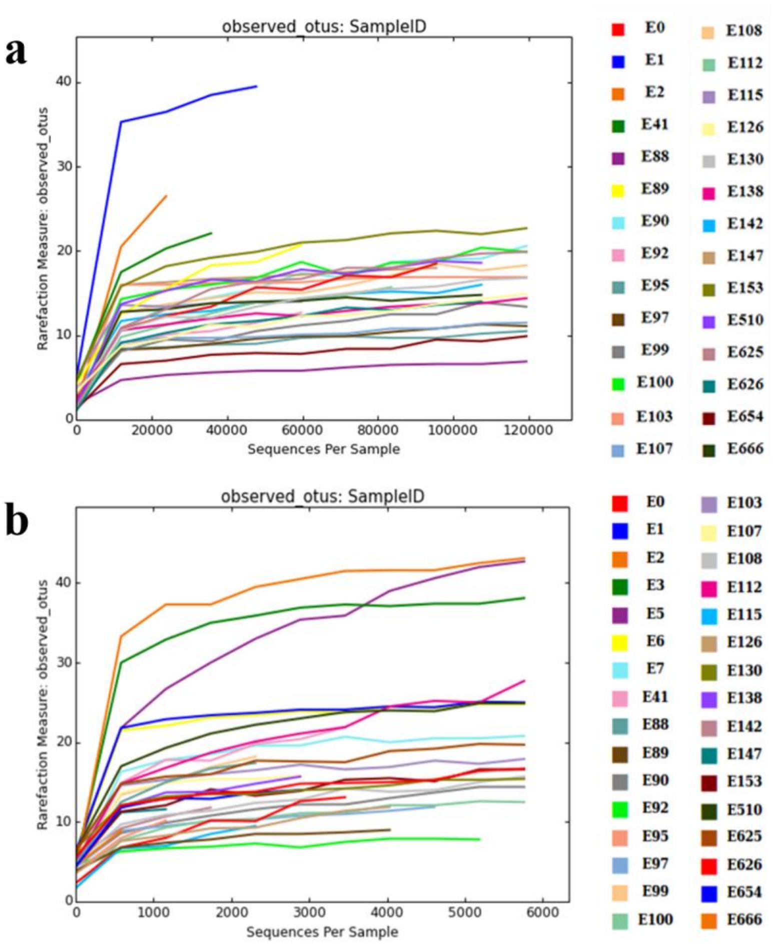

Figure S3. (**a**) Rarefaction plot of 18S v9 eukaryotic reads of each individual at a 100% similarity threshold. (**b**) Rarefaction plot of 16S prokaryotic reads of each individual at a 97% similarity threshold.
